# Supplementary material for: Cross-protective antibodies against common endemic respiratory viruses
Source: Nat Commun. 2023 Feb 13;14:798. doi: 10.1038/s41467-023-36459-3 (PMC9923667; doi:10.1038/s41467-023-36459-3)
Supplement: Supplementary file 1 — Supplementary Information [file 41467_2023_36459_MOESM1_ESM.pdf]

## SUPPLEMENTARY FIGURES AND TABLES

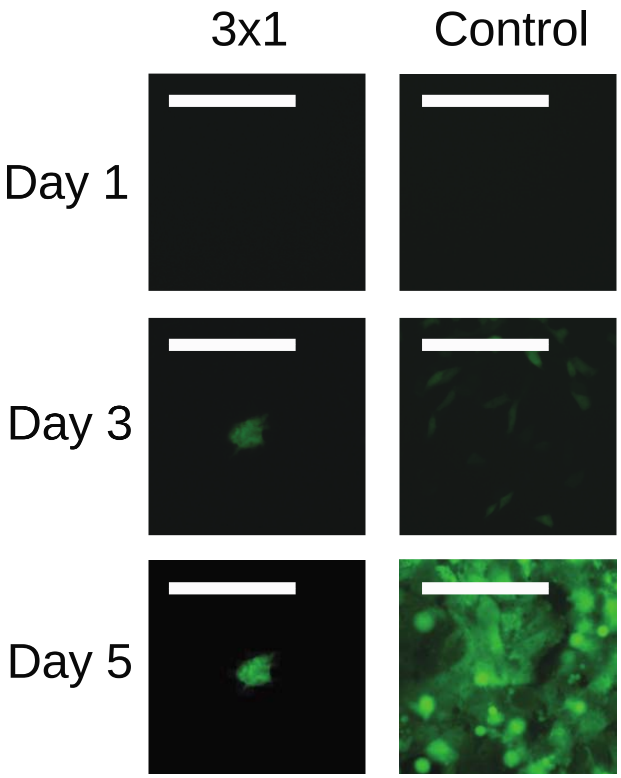


**Supplementary Figure 1. Fusion inhibition assay.** Vero cells were infected with GFP-expressing HPIV3. One-hour post-infection, cells were washed with media to remove un-attached virus. 3x1 mAb (10 µg/mL) or media (control) was added to cells and viral spread and syncytia formation were examined by fluorescent microscopy at days 1, 3, and 5 post-infection. Each condition was performed in duplicate and representative micrographs are shown. Scale bars represent 100 µm.

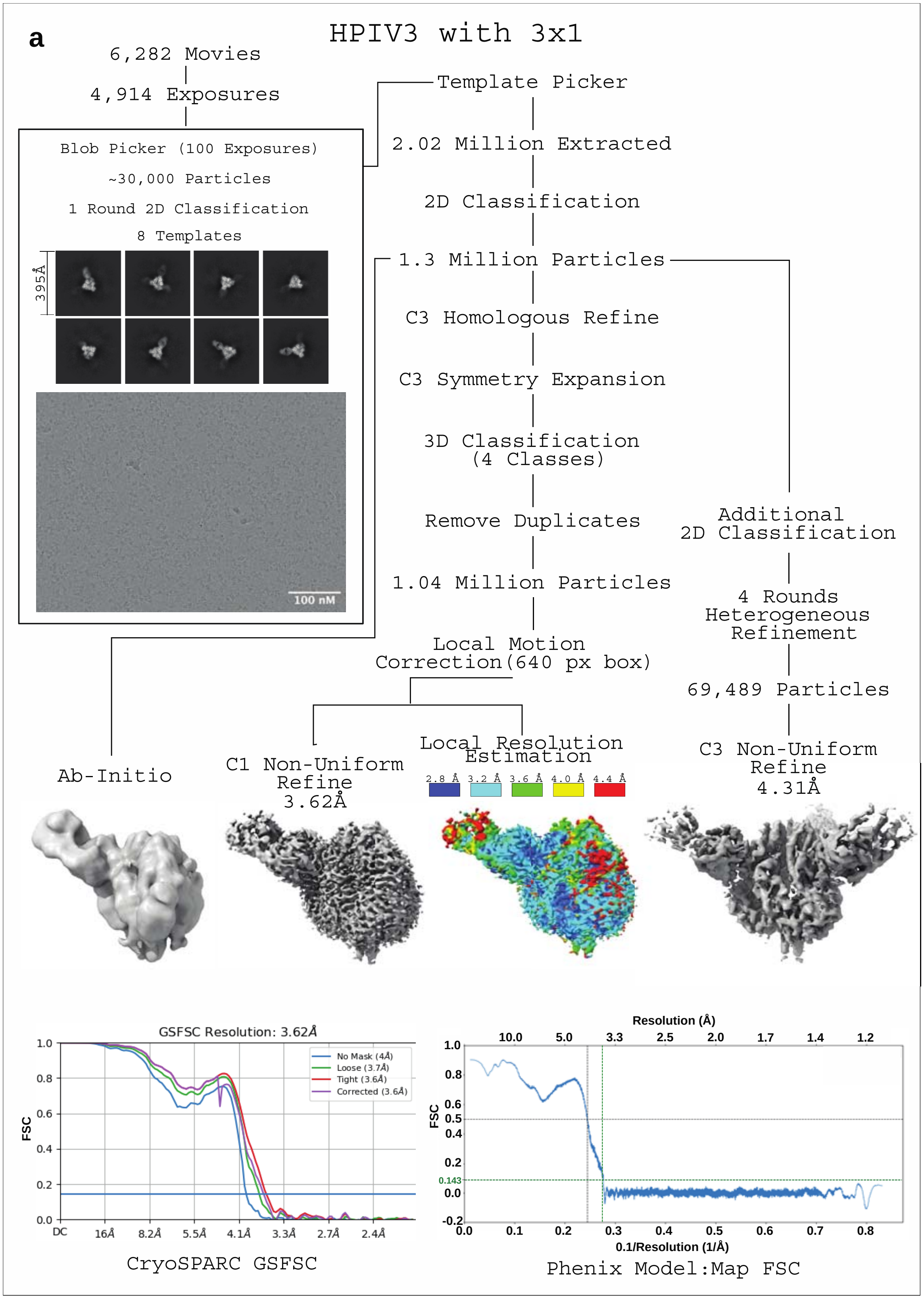
a
HPIV3 with 3x1
6,282 Movies
4,914 Exposures
Blob Picker (100 Exposures)
~30,000 Particles
1 Round 2D Classification
8 Templates
395Å
100 nM
Template Picker
2.02 Million Extracted
2D Classification
1.3 Million Particles
C3 Homologous Refine
C3 Symmetry Expansion
3D Classification (4 Classes)
Remove Duplicates
1.04 Million Particles
Local Motion Correction(640 px box)
Additional 2D Classification
4 Rounds Heterogeneous Refinement
69,489 Particles
Ab-Initio
C1 Non-Uniform Refine 3.62Å
Local Resolution Estimation
2.8 Å 3.2 Å 3.6 Å 4.0 Å 4.4 Å
C3 Non-Uniform Refine 4.31Å
GSFSC Resolution: 3.62Å
No Mask (4Å)
Loose (3.7Å)
Tight (3.6Å)
Corrected (3.6Å)
FSC
DC 16Å 8.2Å 5.5Å 4.1Å 3.3Å 2.7Å 2.4Å
CryoSPARC GSFSC
Resolution (Å)
10.0 5.0 3.3 2.5 2.0 1.7 1.4 1.2
0.143
0.1/Resolution (1/Å)
Phenix Model:Map FSC

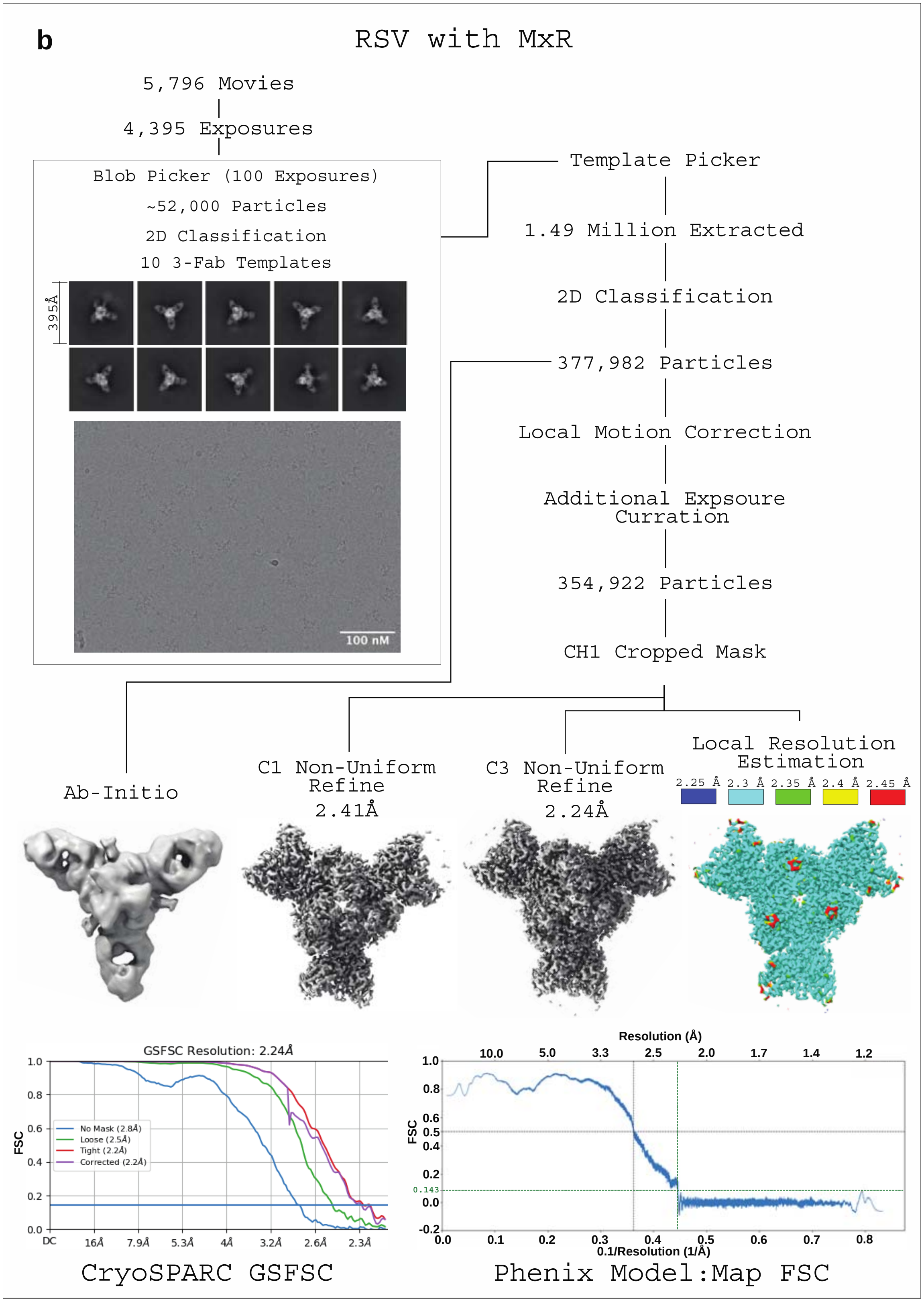


**Supplementary Figure 2. Cryo-EM map processing and resolution.** (**a**) Overall processing pipeline for **Fig. 2** map refinement of the 3x1:HPIV3 complex. (**b**) Overall processing pipeline for **Fig. 4** map refinement of the MxR:RSV complex. Notable maps obtained during processing are shown in miniature. Template creation is shown and boxed, with templates used shown below along with representative micrographs. Final sharp maps are shown with local resolution estimation, CryoSPARC GSFSC, and Phenix mTriage Model:Map FSC at 0.5 and 0.143.

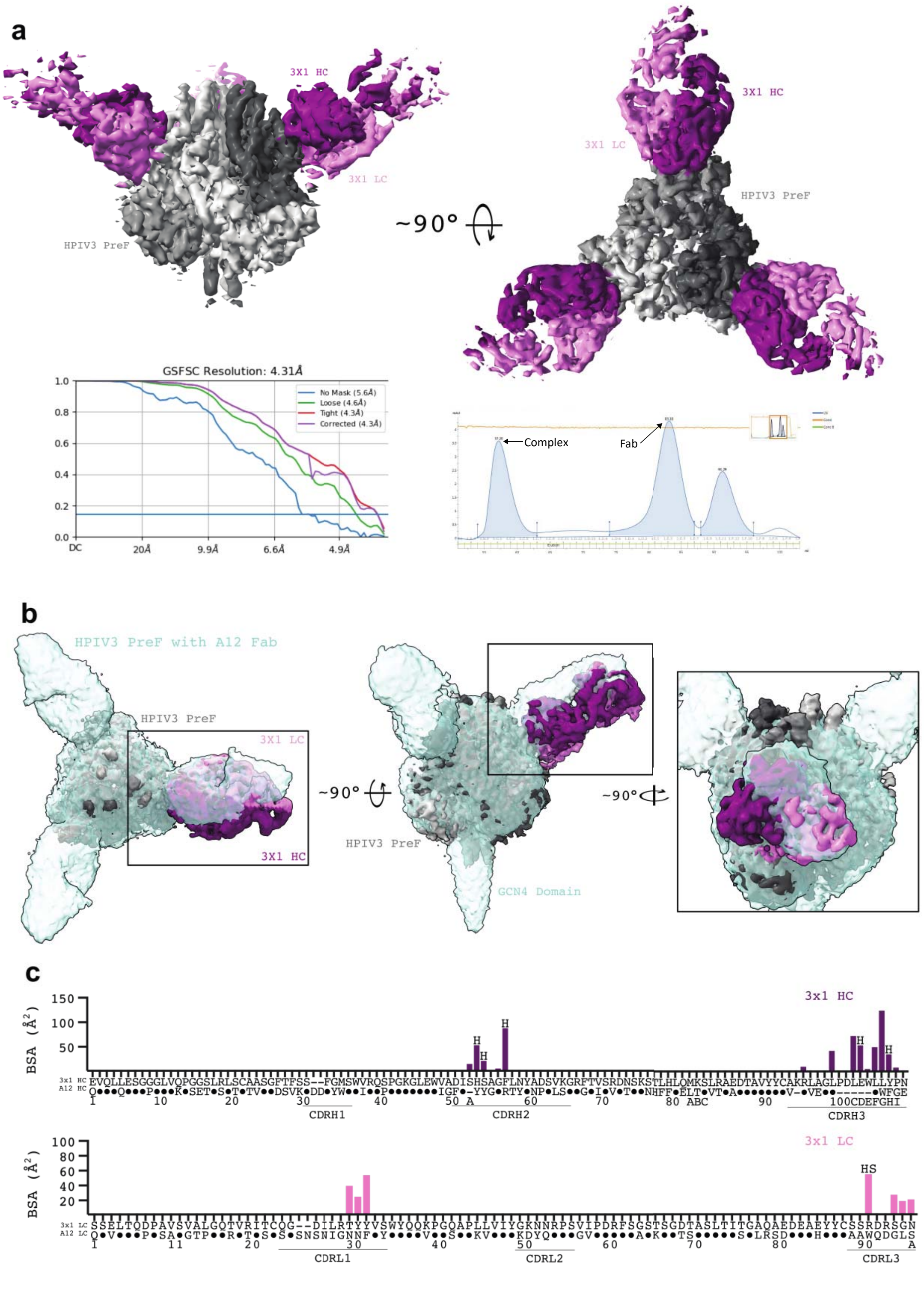


**Supplementary Figure 3. Three Fab map of 3x1:HPIV3 at 4.31 Å resolution and comparison of one Fab 3x1:HPIV3 map to negative stain electron microscopy (nsEM) data of PI3-A12:HPIV3.** (**a**) Top view and side view of the 4.31 Å map of 3x1:HPIV3 with only three Fab bound particles refined using C3 symmetry. CryoSPARC GSFSC curve is shown below. (**b**) Two views of the one Fab 3x1:HPIV3 cryo-EM map and PI3-A12:HPIV3 nsEM map are shown aligned. PI3-A12 is abbreviated A12. Labels show regions of interest in the corresponding color. Zoomed in view shows deviation in the binding orientation of 3x1 versus PI3-A12**.** (**c**) BSA plots show residues of 3x1 which interact with the HPIV3 preF protomer, atop a sequence alignment with PI3-A12. Dots indicate conserved residues, dashes indicate gaps in the aligned sequence.

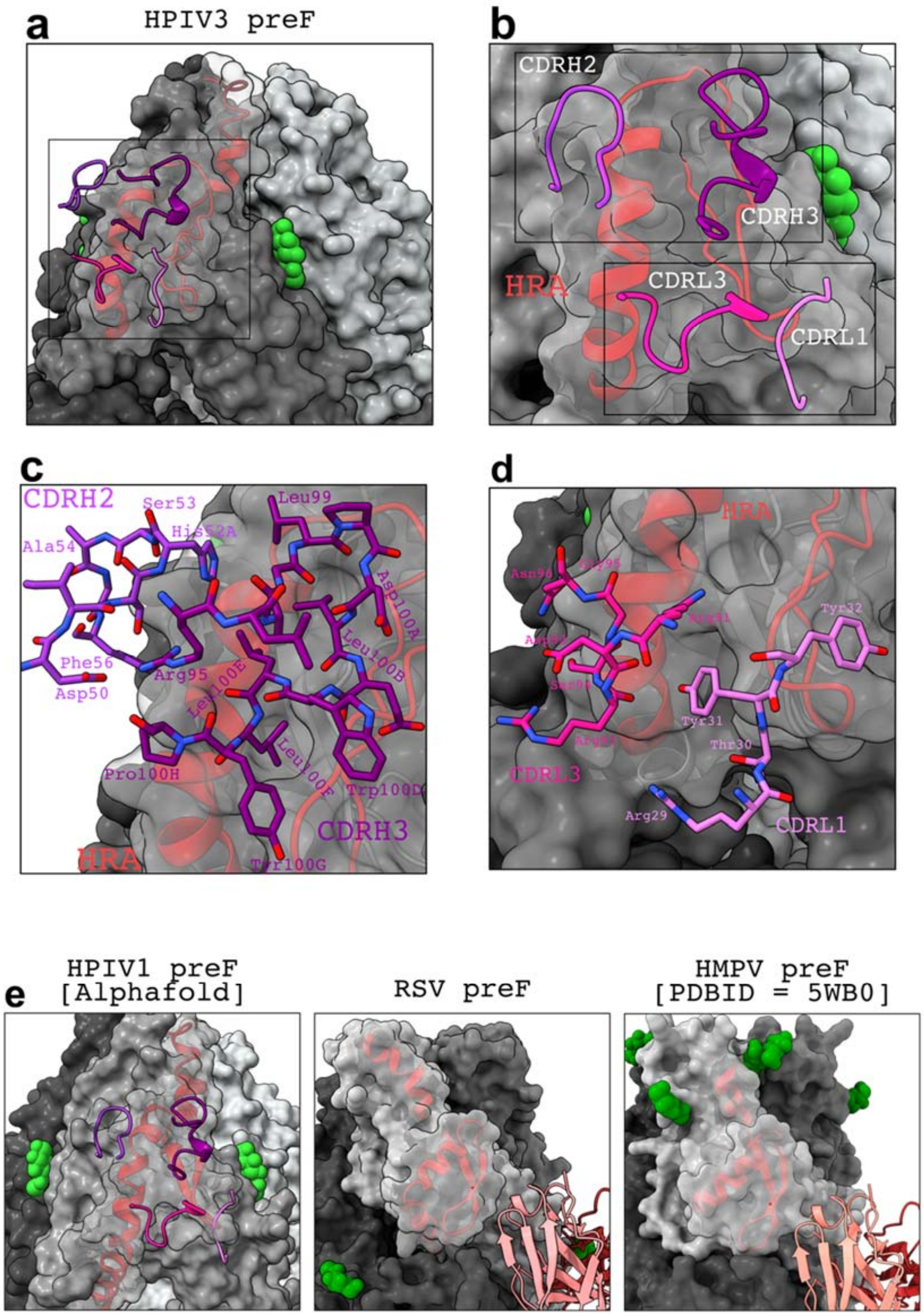


**Supplementary Figure 4. Heptad Repeat A (HRA) of HPIV3 bound to 3x1, with representative views of the HRA region in HPIV1, RSV, and HMPV.** (**a**) HPIV3 preF shown with the HRA colored in red with a transparent surface. 3x1 CDRs are shown in their colors from **Figure 2**. (**b**) View of the 3x1 CDRs in contact with the HRA helix of HPIV3 preF. CDRs are labeled and colored distinctly, HRA is shown in red, and glycans are shown in green. (**c**) In-depth image of the CDRH2 and H3 bound to the HRA region. (**d**) In-depth image of the CDRL1 and L3 bound to the HRA region. (**e**) The HRA region of HPIV1, RSV, and HMPV preF are shown in a similar manner to previous panels. HPIV1 is an Alphafold generated model aligned to HPIV3 protomer map density with CDRs superimposed. RSV preF is the model published in this paper. HMPV preF is the model from the listed structure, aligned to the RSV preF trimer (RMSD = 1.461 Å over 1136 Cα).

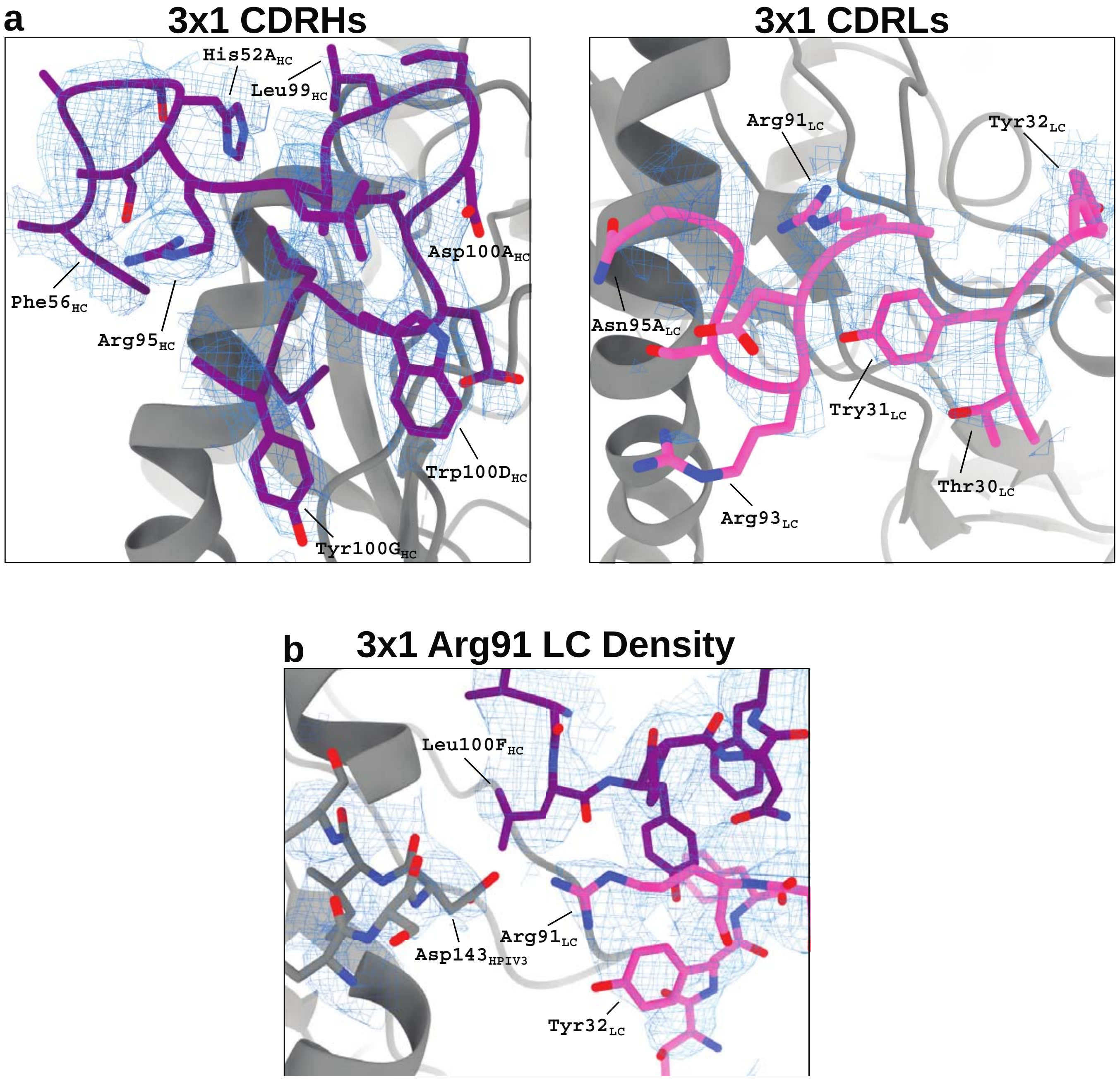


**Supplementary Figure 5. Map density at HPIV3 CDRs and light chain residue Arg 91.** (**a**) Structure of 3x1:HPIV3 shown in cartoon representation. Image has been depth clipped and only sidechains are shown in sticks for clarity. Cryo-EM map density from CryoSPARC sharpened map shown at ~5 sigma in blue mesh. HPIV3 preF is shown in shades of grey. (**b**) Density specifically showing light chain residue Arg 91. Adjacent interacting residues include HC Leu 100F, LC Tyr 31 and HPIV3 preF Asp 143.

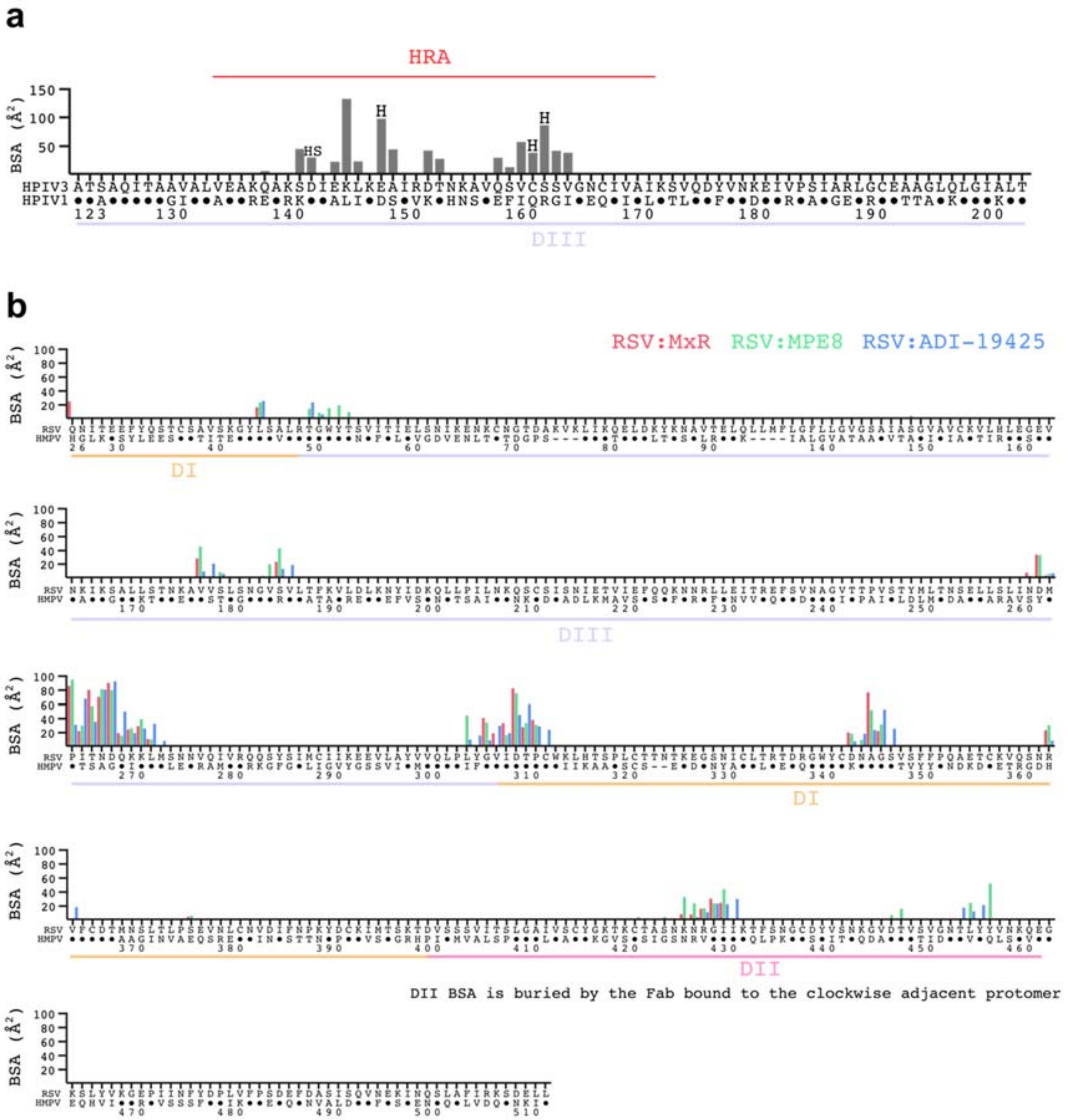


**Supplementary Figure 6. Per residue BSA of interacting HPIV3 and RSV preF residues.** (**a**) BSA plot of HPIV3 residues which contact 3x1; this is a complementary plot to **Fig. 2c**. Only residues 123-204 – all within domain III – contact 3x1 Fab. Below the HPIV3 sequence, the HPIV1 sequence is aligned. (**b**) BSA plot showing RSV residue interactions with mAbs in MxR:RSV, MPE8:RSV, and ADI-19425:RSV; this data is complementary to **Fig. 4b**. The sequences of RSV and HMPV F are aligned below. Below that, colored bars indicate the structural domains of RSV using the same colors as **Fig. 4a**.

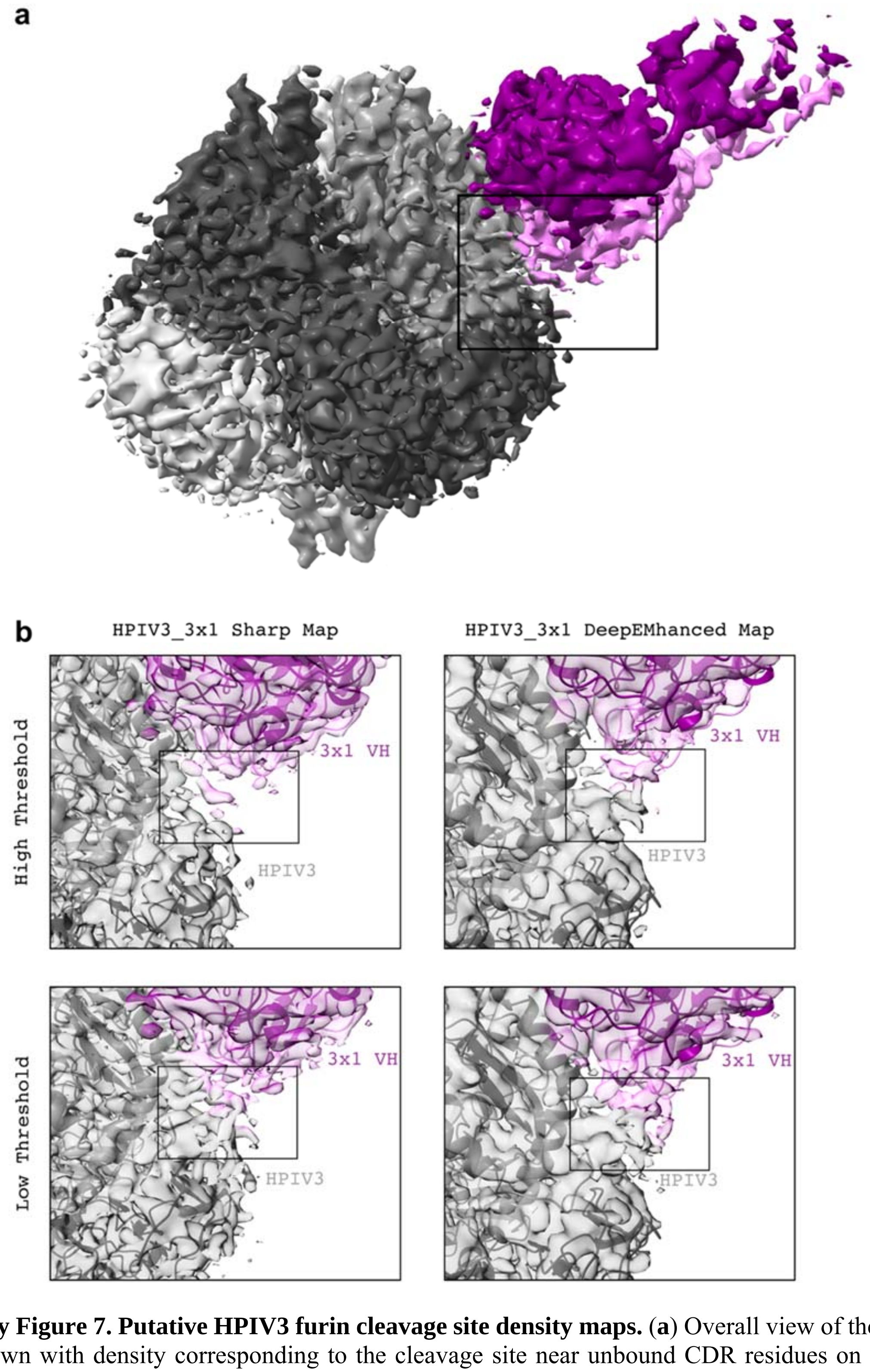


**Supplementary Figure 7. Putative HPIV3 furin cleavage site density maps.** (**a**) Overall view of the 3x1:HPIV3 complex is shown with density corresponding to the cleavage site near unbound CDR residues on 3x1. (**b**) The region of interest is shown as both the sharp map and DeepEMhanced map at both high and low thresholds, with the high threshold approximately the normal structure building level, ~5 sigma. The density has a resemblance to a sheet-turn-sheet motif which would be appropriate for the C-terminus of the F2 protein product following furin cleavage.

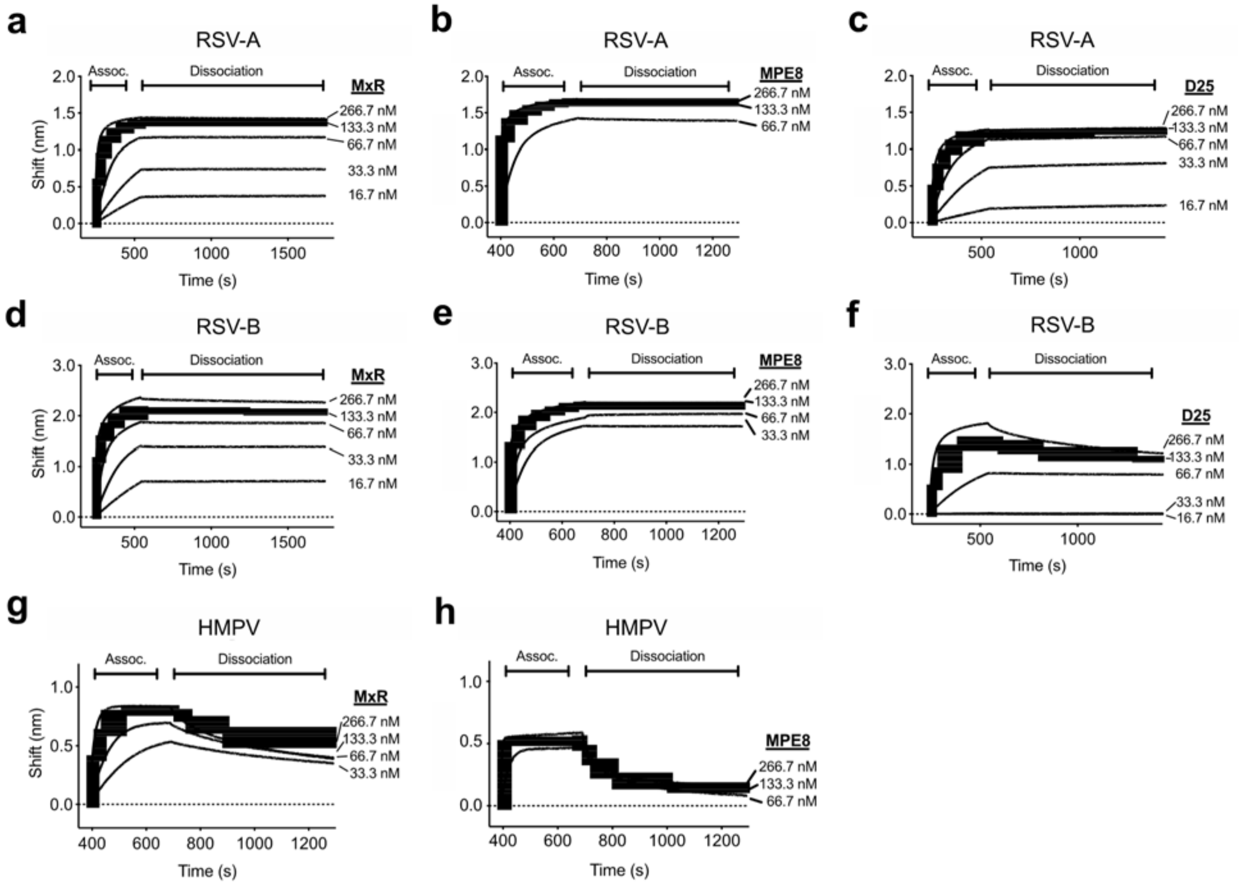


**Supplementary Figure 8. Binding kinetics of cross-neutralizing monoclonal antibodies.** Apparent affinity ($K_D$) of MxR (**a, d, g**), MPE8 (**b, e, h**), and D25 (**c, f**) at concentrations ranging from 16.7-266.7 nM to RSV-A (**a-c**), RSV-B (**d-f**), and HMPV (**g-h**) preF was determined by BLI. Penta-His capture sensors were loaded with His-tagged preF proteins. Association with MxR, MPE8, and D25 was then measured for 300 s followed by dissociation for at least 600 s. All measurements are normalized against an isotype control antibody.

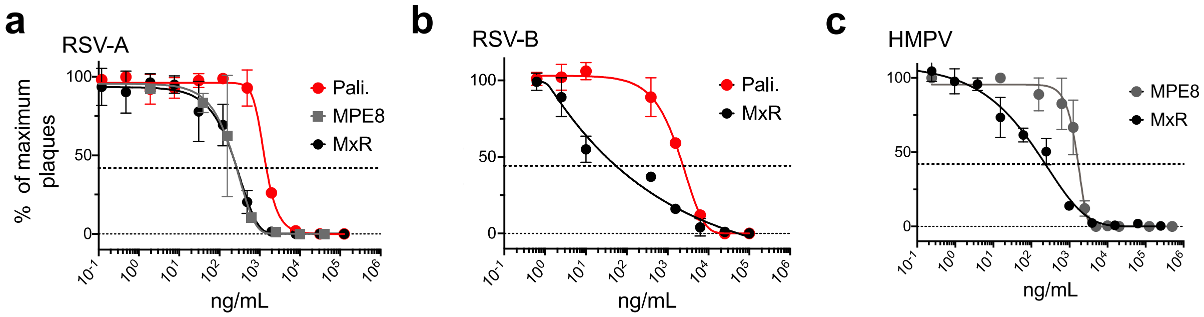


**Supplementary Figure 9. Neutralization potency *in vitro* of cross-neutralizing monoclonal antibodies.** Vero cells were infected with RSV-A (**a**), RSV-B (**b**), or HMPV (**c**) in the presence of serial dilutions of palivizumab (abbreviated Pali.), MPE8, or MxR. The dotted midline indicates the $PRNT_{60}$. Data points represent the mean from three independent experiments with each experiment consisting of two technical replicates. Error bars indicate standard deviation.

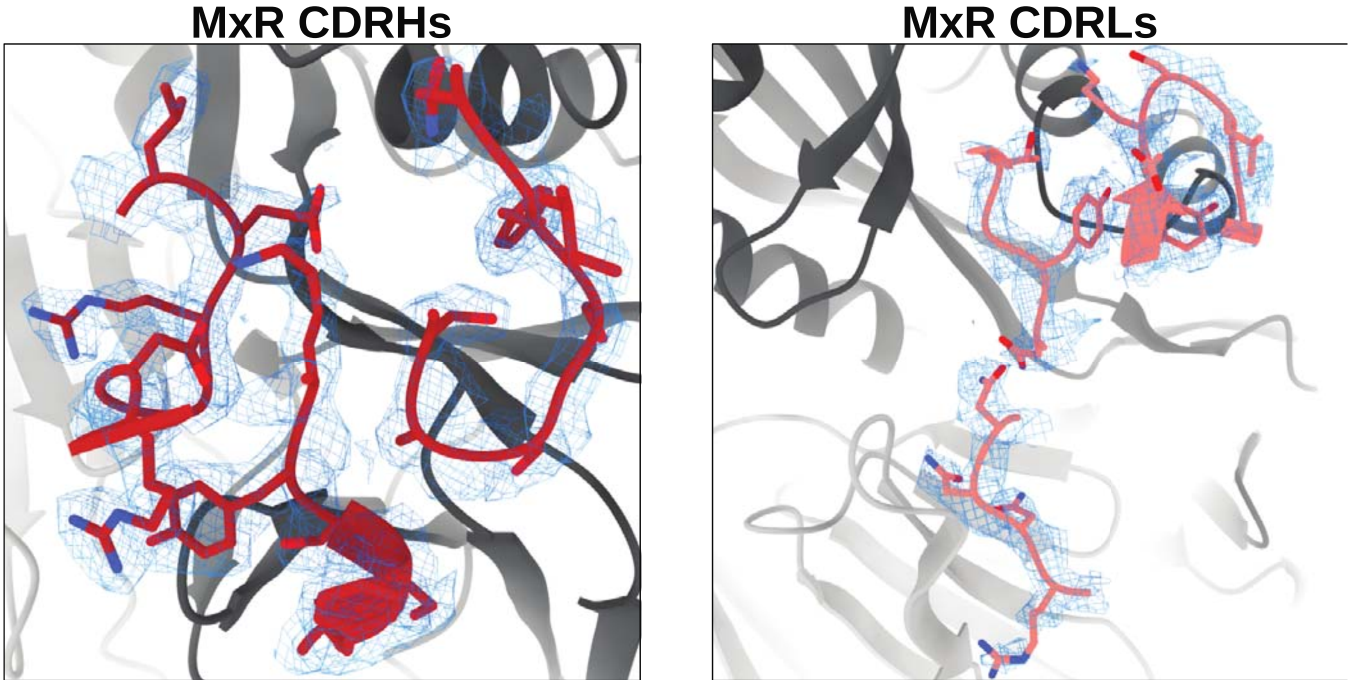


**Supplementary Figure 10. Map density at MxR CDRs.** Structure of MxR:RSV shown in cartoon representation. Image has been depth clipped and only sidechains are shown in sticks for clarity. Cryo-EM map density from CryoSPARC sharpened map shown at ~5 sigma in blue mesh. RSV preF is shown in shades of grey.

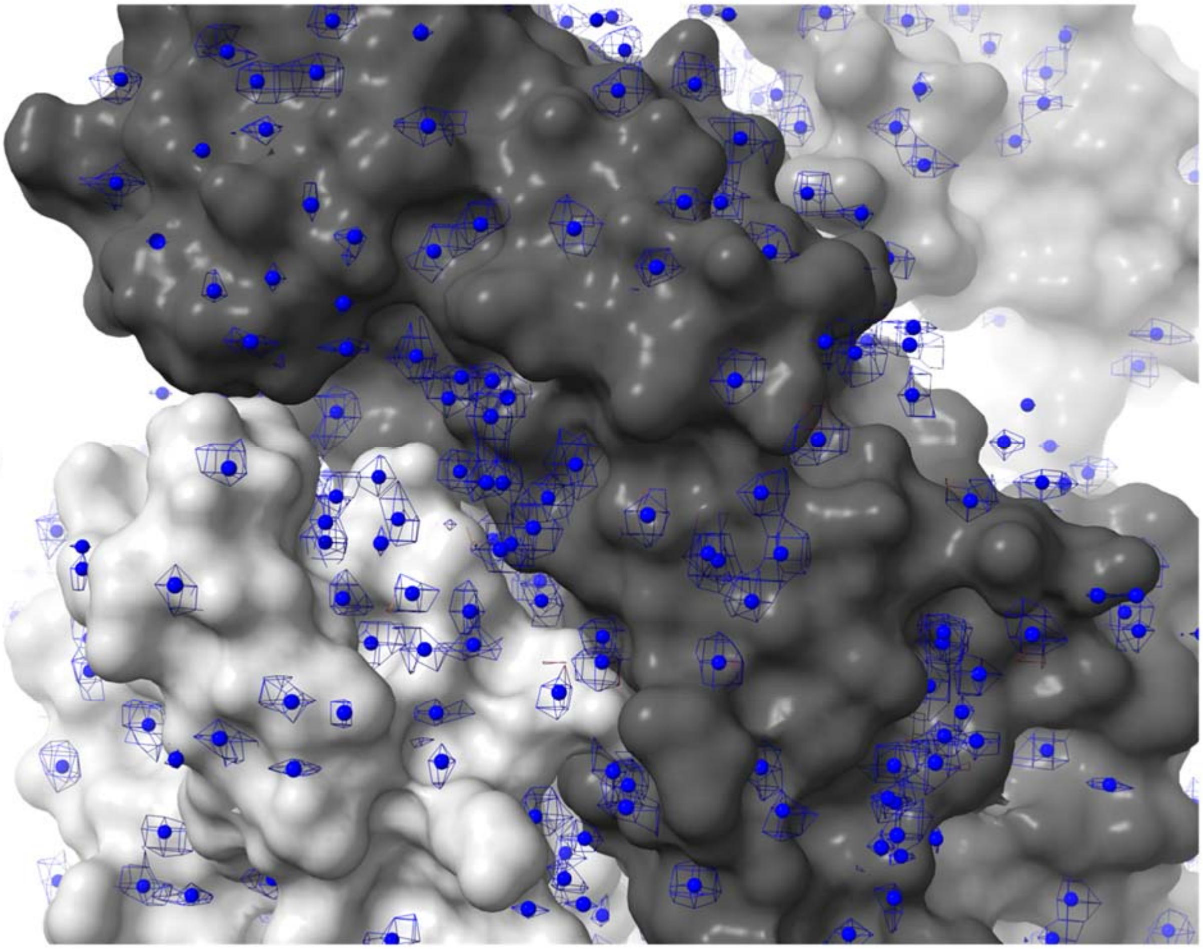

**Supplementary Figure 11. Water molecules and density at the MxR binding site in MxR:RSV.** Structure of MxR:RSV shown with MxR removed to show binding site. Water molecules shown as blue dots with surrounding electron map density shown in blue mesh. RSV preF is shown in shades of grey.

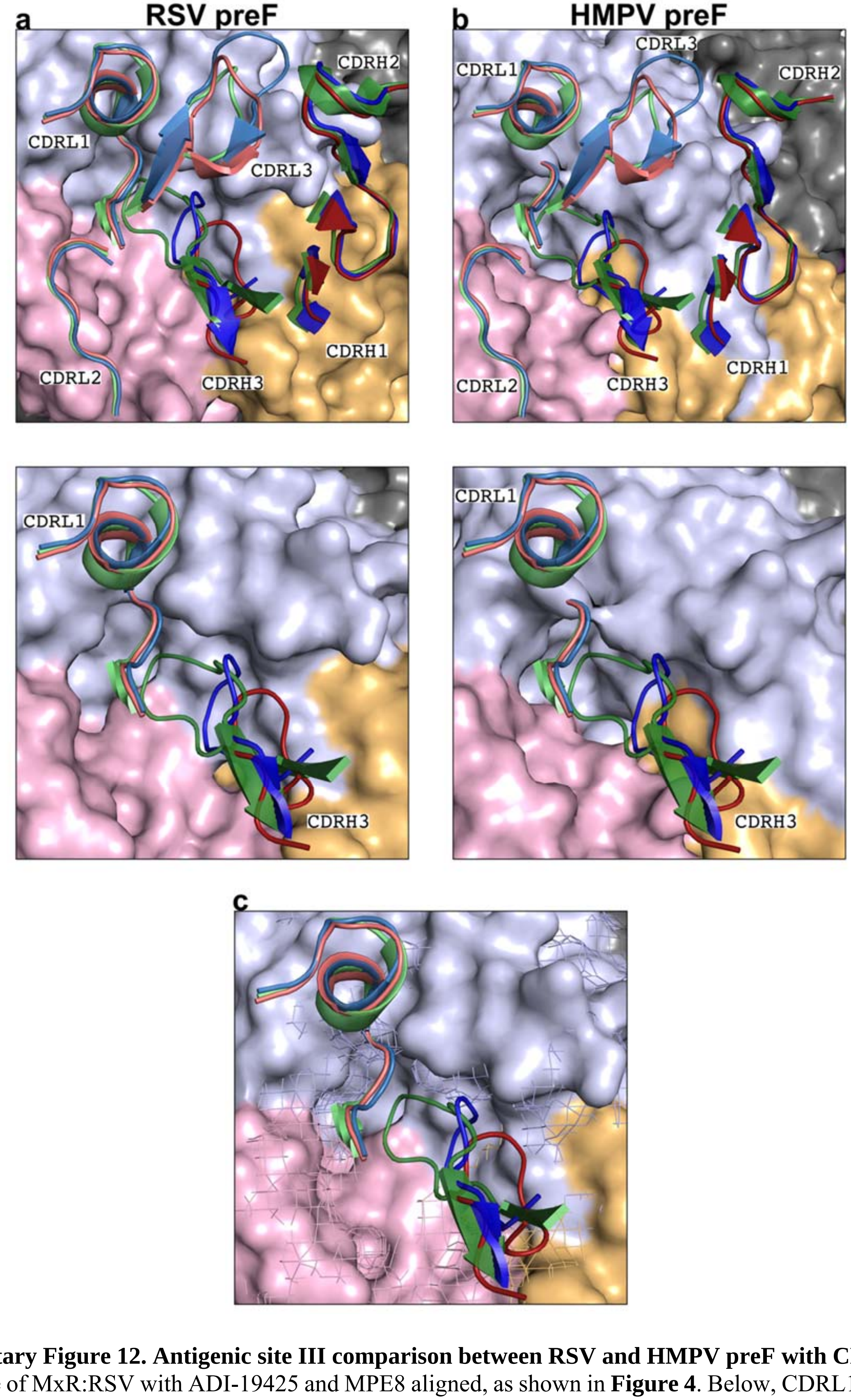


**Supplementary Figure 12. Antigenic site III comparison between RSV and HMPV preF with CDR overlays.** (**a**) Structure of MxR:RSV with ADI-19425 and MPE8 aligned, as shown in **Figure 4**. Below, CDRL1 and CDRH3 are shown in greater detail. (**b**) MxR, ADI-19425, and MPE8 CDRs are shown with HMPV preF (PDB ID 5WB0) aligned to RSV preF (RMSD 1.461 Å over 1136 Cα). Coloring is consistent with HMPV structural domains. Below, CDRL1 and CDRH3 are shown in detail. (**c**) The lower image of panel (**a)** is shown with aligned HMPV in mesh.

**Supplemental Table 1.** 3x1:HPIV3 and MxR:RSV cryo-EM structure statistics

| | **HPIV3 with 3x1** | **RSV with MxR** |
|---|---|---|
| **Data collection** | | |
| Microscope | FEI Titan Krios | FEI Titan Krios |
| Voltage (kV) | 300 | 300 |
| Electron Dose (e-/ $Å^2$) | 50 | 50 |
| Detector | Gatan K3 DED | Gatan K3 DED |
| Pixel Size (Å /px) | 1.02885 | 1.02885 |
| Defocus Range (μm) | -0.5 to -3.5 | -1.0 to -3.0 |
| Collection Tilt (°) | 30 | 0 |
| Magnification | 92,000x | 92,000x |
| | | |
| **Reconstruction** | | |
| Software | cryoSPARC v3.3.1 | cryoSPARC v3.3.1 |
| Selected Micrographs | 4914 | 3900 |
| Selected Particles | 1,036,244 | 354,922 |
| Symmetry | C1 | C3 |
| Box Size (px) | 360 | 384 |
| Resolution (Å) ($FSC_{0.143}$) | 3.62 | 2.24 |
| | | |
| **Refinement** | | |
| Software | Phenix 1.19.2-4158 | Phenix 1.19.2-4158 |
| Map B factor ($Å^2$) | -153.1 | -71.4 |
| No. atoms | 11,617 | 16,921 |
| Protein | 11,347 | 15,270 |
| Water | - | 1471 |
| Ligand | 270 | 180 |
| Mean B-factor ($Å^2$) | | |
| Protein | 125.86 | 31.47 |
| Water | - | 37.33 |
| Ligand | 126.38 | 64.52 |
| RMS bond length (Å) | 0.004 | 0.004 |
| RMS bond angle (°) | 0.816 | 0.632 |
| | | |
| **Validation** | | |
| MolProbity | 2.20 | 1.50 |
| Clashscore | 15.71 | 4.85 |
| CaBLAM Outliers (%) | 4.84 | 0.00 |
| EMRinger | 1.55 | 4.61 |
| Rotamer Outliers (%) | 0.15 | 1.42 |
| Ramachandran | | |
| Favored (%) | 91.56 | 97.31 |
| Disallowed (%) | 0.07 | 0.00 |
| | | |
| **PDB ID** | 8DG8 | 8DG9 |
| **EMDB ID** | 27418 | 27419 |

Bolded text indicates section headers

**Antibody protein sequences**

>MxR heavy chain variable region
EVQVVESGGGLVKPGGSLRLSCAASGFPFSSYKMDWVRQAPGKGLEWVSSISASGSYINYADSVKGRFT
ISRDNAKNSLYLQMKSLRADDTAVYFCARDGGRELSPFEKWGQGILVTVSS

>MxR-01 light chain variable region
QSVLTQPPSVSGAPGQRVTISCTGTNSNIGTGYDVHWYQQLPGTAPKVVLFDNNNRPSGVPDRFSGSKSG
TSAALAITGLQAEDEAVYYCQSYDKSLGGWVFGGGTKLTVL

>3x1 heavy chain variable region
EVQLLESGGGLVQPGGSLRLSCAASGFTFSSFGMSWVRQSPGKGLEWVADISHSAGFLNYADSVKGRFT
VSRDNSKSTLHLQMKSLRAEDTAVYYCAKRLAGLPDLEWLLYPNFLDHWGQGTLVTVSS

>3x1 light chain variable region
SSELTQDPAVSVALGQTVRITCQGDILRTYYVSWYQQKPGQAPLLVIYGKNNRPSVIPDRFSGSTSGDTA
SLTITGAQAEDEAEYYCSSRDRSGNHVLFGGGTKLTVL
